# Theaflavin-3,3′-Digallate Enhances the Inhibitory Effect of Cisplatin by Regulating the Copper Transporter 1 and Glutathione in Human Ovarian Cancer Cells

**DOI:** 10.3390/ijms19010117

**Published:** 2018-01-02

**Authors:** Haibo Pan, Eunhye Kim, Gary O. Rankin, Yon Rojanasakul, Youying Tu, Yi Charlie Chen

**Affiliations:** 1 Department of Tea Science, Zhejiang University, Hangzhou 310058, China; 0801080115@cau.edu.cn (H.P.); ehkim@zju.edu.cn (E.K.); 2 College of Science, Technology and Mathematics, Alderson Broaddus University, Philippi, WV 26416, USA; 3 Department of Biomedical Sciences, Joan C. Edwards School of Medicine, Marshall University, Huntington, WV 25755, USA; rankin@marshall.edu; 4 Department of Pharmaceutical Sciences, West Virginia University, Morgantown, WV 26506, USA; yrojan@hsc.wvu.edu

**Keywords:** theaflavin-3,3′-digallate, cisplatin, ovarian cancer, copper transporter 1, glutathione

## Abstract

Ovarian cancer has the highest fatality rate among the gynecologic cancers. The side effects, high relapse rate, and drug resistance lead to low long-term survival rate (less than 40%) of patients with advanced ovarian cancer. Theaflavin-3,3′-digallate (TF3), a black tea polyphenol, showed less cytotoxicity to normal ovarian cells than ovarian cancer cells. We aimed to investigate whether TF3 could potentiate the inhibitory effect of cisplatin against human ovarian cancer cell lines. In the present study, combined treatment with TF3 and cisplatin showed a synergistic cytotoxicity against A2780/CP70 and OVCAR3 cells. Treatment with TF3 could increase the intracellular accumulation of platinum (Pt) and DNA-Pt adducts and enhanced DNA damage induced by cisplatin in both cells. Treatment with TF3 decreased the glutathione (GSH) levels and upregulated the protein levels of the copper transporter 1 (CTR1) in both cells, which led to the enhanced sensitivity of both ovarian cancer cells to cisplatin. The results imply that TF3 might be used as an adjuvant to potentiate the inhibitory effect of cisplatin against advanced ovarian cancer.

## 1. Introduction

Ovarian cancer is the tenth most common cancer and the fifth leading cause of cancer death among women in the United States [1]. Ovarian cancer causes a higher death rate than any other gynecologic cancer, leading to approximately 5% of cancer deaths among women. The conventional treatment for advanced ovarian cancer is surgical resection of the tumor mass followed by platinum based-chemotherapy [2]. Although most patients with advanced ovarian cancer respond well to the first-line conventional treatment, 70% of the patients relapse within 18 months of the treatment [3]. Moreover, the used chemotherapeutic agents often become ineffective to relapsing patients. The high relapse rate and drug resistance lead to low long-term survival rate (less than 40%) of patients with advanced ovarian cancer [4].

Cisplatin is the first platinum-based drug approved for ovarian cancer and one of the most active chemotherapy agents for the treatment of ovarian cancer. In cells, DNA is the primary target of cisplatin. The mechanism of its antitumor action is related to crosslinking the purine in the DNA chain forming intra-strand and inter-strand cross-linking [5]. Multiple mechanisms are involved in the antitumor effect of cisplatin, of which the most known mode of action is to activate DNA damage response and induce mitochondrial apoptosis triggered by generating DNA lesions [6]. Cisplatin resistance attenuates its antitumor effect and limits its clinical employment. Mechanisms of cisplatin resistance are complex, which is always accompanied by reduced intracellular accumulation, increased sequestration, increased DNA lesion repair, and alterations in apoptotic signaling pathways [7]. Several studies demonstrated that copper transporter 1 (CTR1) is a major transmembrane protein involving the uptake of cisplatin. Enhanced expression of CTR1 sensitized ovarian cancer cells to cisplatin by increasing cisplatin uptake [8]. CTR1 defect embryonic fibroblasts were more resistant to cisplatin than the wild-type counterparts [9]. Pre-treatment with copper was able to protect cells from cisplatin cytotoxicity, whereas copper chelator led to increased cisplatin uptake and enhanced cisplatin cytotoxicity [10,11]. Multidrug resistance protein 2 (MRP2), copper-transporting P-type ATPase (ATP7A and ATP7B), and glutathione (GSH) are the major proteins mediating cisplatin resistance by increasing cisplatin efflux. Overexpression of MRP2 led to cisplatin resistance in melanoma cells by decreasing the formation of cisplatin-DNA adducts [12]. Increased expression of ATP7A led to cisplatin resistance in ovarian cancer cells [13]. Moreover, clinical study indicated that ATP7B was a cisplatin-resistant marker in ovarian cancer [14]. Cisplatin bound stably to GSH in cytoplasm before it was transferred into the nucleus and mitochondria, which limited the amount of cisplatin reacting with DNA [15]. In addition, the GSH-cisplatin complexes were readily exported by MRP2 [16]. Thus, cisplatin transporters and GSH play an important role in cisplatin resistance. Targeting cisplatin transporters and GSH might be an effective intervention to overcome cisplatin resistance.

TF3 is a polyphenolic compound extracted from black tea and is formed via the co-oxidation of (−)-epigallocatechin gallate (EGCG) and (−)-epicatechin gallate during black tea production [17]. TF3 has shown potent antioxidant [18], anti-inflammatory [19], and anticancer properties [20,21,22]. We have previously reported that TF3 inhibited human ovarian cancer cells by inducing apoptosis and cell cycle arrest [23] and by suppressing angiogenesis [24]. In the present study, we investigated whether TF3 could potentiate the inhibitory effect of cisplatin against human ovarian cancer cell lines.

## 2. Results

### 2.1. The Synergistic Inhibitory Effect of TF3 and Cisplatin Against Ovarian Cancer Cells

**Figure 1:**
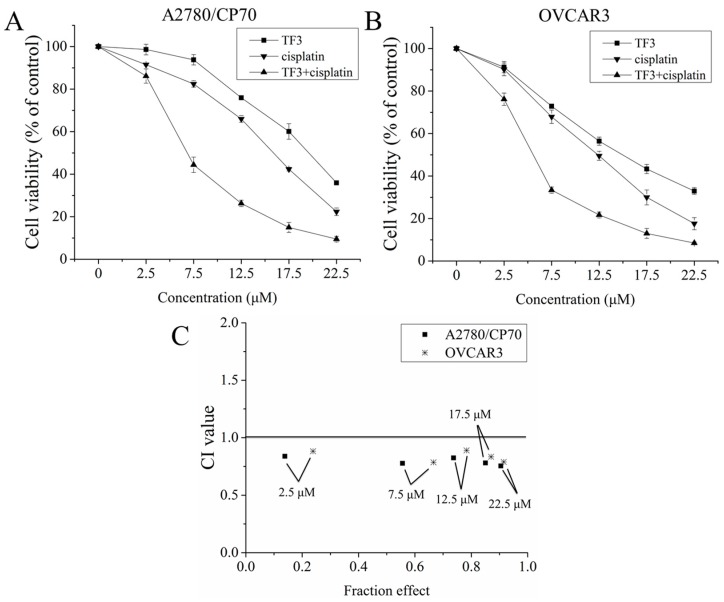
The synergistic inhibitory effect of TF3 and cisplatin against ovarian cancer A2780/CP70 and OVCAR3 cells. (**A**) The cell viability of A2780/CP70 cells treated with TF3, cisplatin, or their combination at a ratio of 1:1 (μM:μM) at the designated concentrations; (**B**) the cell viability of OVCAR3 cells treated with TF3, cisplatin, or their combination at a ratio of 1:1 (μM:μM) at the designated concentrations; (**C**) the CI values of TF3 and cisplatin combination treatment against both cells at a constant ratio of 1:1 (μM:μM) at the designated concentrations were less than 1.0, indicating synergism. Results are expressed as mean ± SD from three independent experiments.

To determine the synergistic inhibitory effect of TF3 and cisplatin against ovarian cancer A2780/CP70 and OVCAR3 cells, we evaluated the combination index (CI) of TF3 and cisplatin at a constant ratio of 1:1 (μM:μM). The viability of both cells treated with TF3, cisplatin, and their combination was determined using the MTS assay. As shown in Figure 1A,B, TF3, cisplatin, and the combination treatment decreased the viability of both cells in a dose-dependent manner. CI values were calculated using CalcuSyn software. As shown in Figure 1C, the CI values of combination treatment at the designated concentration were less than 1.0, which indicated that the inhibitory effect of TF3 and cisplatin combination treatment against ovarian cancer cells was synergistic.

### 2.2. TF3 Increases Intracellular Accumulation of Total Pt and DNA-Pt Adducts

**Figure 2:**
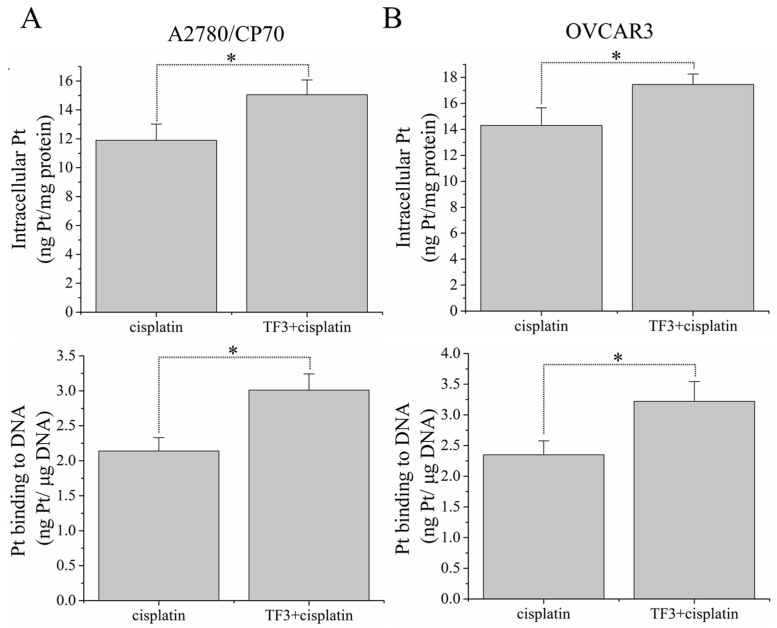
Effects of TF3 on the accumulation of Pt and DNA-Pt adducts in A2780/CP70 and OVCAR3 cells. Cells were treated with 7.5 μM cisplatin or 7.5 μM combined TF3 and cisplatin for 6 h followed by the ICP-MC assay. (**A**) Effects of TF3 on the accumulation of Pt and DNA-Pt adducts in A2780/CP70 cells; (**B**) effects of TF3 on the accumulation of Pt and DNA-Pt adducts in OVCAR3 cells. Data represent means ± SD of three independent experiments. Significant differences among different treatments are marked with * (*p* < 0.05).

To investigate whether the synergistic inhibitory effect of TF3 and cisplatin against ovarian cancer A2780/CP70 and OVCAR3 cells resulted from changes in intracellular Pt accumulation, the total Pt and DNA-Pt adducts in cells were measured by ICP-MS. As shown in Figure 2, treatment with TF3 significantly increased the accumulation of total Pt and DNA-Pt adducts in A2780/CP70 and OVCAR3 cells (*p* < 0.05). The total Pt accumulated in A2780/CP70 cells treated with 7.5 μM cisplatin for 6 h was 11.90 ± 1.12 ng Pt/mg protein, which was significantly lower than that (15.05 ± 1.02 ng Pt/mg protein) in cells treated with 7.5 μM TF3 and cisplatin. The Pt-DNA adducts accumulated in A2780/CP70 cells treated with 7.5 μM cisplatin for 6 h was 2.14 ± 0.19 ng Pt/μg DNA, which was significant lower than that (3.01 ± 0.23 ng Pt/μg DNA) in cells treated with 7.5 μM TF3 and cisplatin. The total Pt accumulated in OVCAR3 cells treated with 7.5 μM cisplatin for 6 h was 14.32 ± 1.36 ng Pt/mg protein, which was significant lower than that (17.45 ± 0.82 ng Pt/mg protein) in cells treated with 7.5 μM TF3 and cisplatin. The Pt-DNA adducts accumulated in OVCAR3 cells treated with 7.5 μM cisplatin for 6 h was 2.35 ± 0.22 ng Pt/μg DNA, which was significantly lower than that (3.22 ± 0.32 ng Pt/μg DNA) in cells treated with 7.5 μM TF3 and cisplatin. Therefore, treatment with TF3 could increase the accumulation of Pt in both cells and nuclei, which led to the synergistic effect of TF3 and cisplatin against ovarian cancer cells.

### 2.3. TF3 Enhanced DNA Damage Induced by Cisplatin in Ovarian Cancer Cells

**Figure 3:**
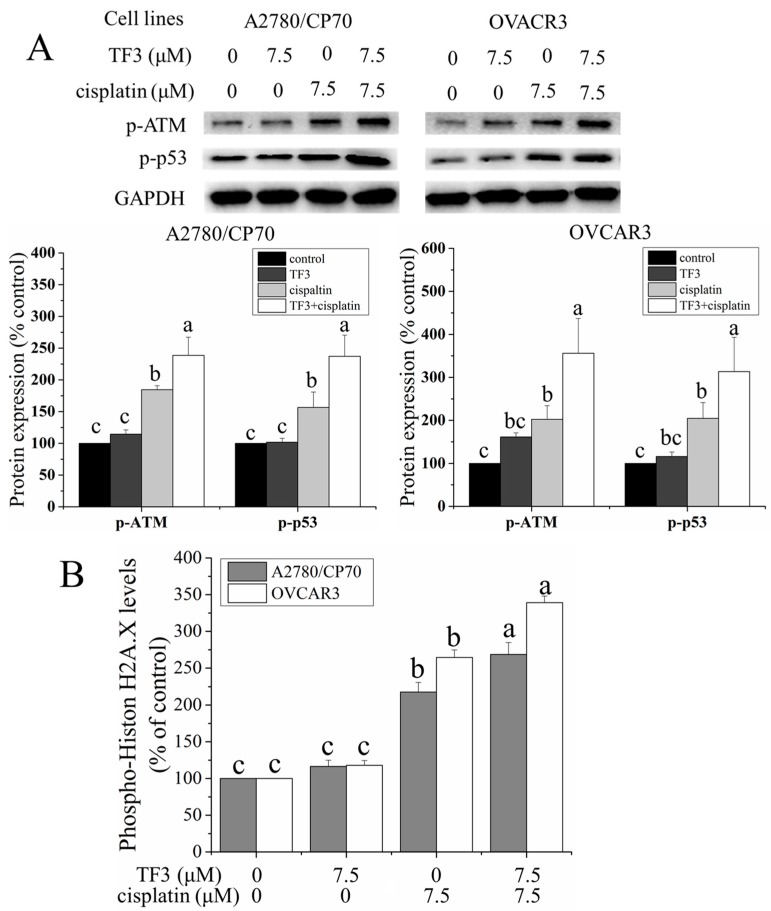
Treatment with 7.5 μM TF3 enhanced DNA damage induced by 7.5 μM cisplatin in ovarian cancer A2780/CP70 and OVCAR3 cells. (**A**) The effect of TF3, cisplatin and combination treatment of the protein levels of p-ATM and p-p53 in ovarian cancer cells were determined by Western blot analysis; (**B**) the effect of TF3, cisplatin and combination treatment of the protein level of p-Histon H2A.X in ovarian cancer cells was determined by ELISA assay. Results are expressed as mean ± SD from three independent experiments. Significant differences among different treatments are marked with different letters (*p* < 0.05).

Cisplatin is known to exert antitumor effect mainly by inducing DNA damage. DNA damage levels in ovarian cancer cells were determined by Western blot analysis and enzyme-linked immunosorbent assay (ELISA) assay. Ataxia telangiectasia mutated kinase (ATM), a serine/threonine kinase, is a key sensor and transducer of DNA damage signals. ATM is phosphorylated on Ser1981 induced by DNA damage and phosphorylates series of downstream signaling molecules. The p53 protein phosphorylated by ATM at Ser15 in response to DNA damage. As shown in Figure 3A, treatment with 7.5 μM TF3 had no significant effect on the protein levels of p-ATM (Ser1981) and p-p53 (Ser15) (*p* > 0.05). Treatment with 7.5 μM cisplatin significantly upregulated the protein levels of p-ATM (Ser1981) and p-p53 (ser15) (*p* < 0.05). The protein levels of p-ATM (Ser1981) and p-p53 (Ser15) were significantly higher in both cells subjected to combination treatment compared to the untreated control cells and cells treated with either agent alone (*p* < 0.05). The phosphorylation of Histone H2A.X at Ser139 is a marker of DNA damage, which was detected using ELISA assay. As shown in Figure 3B, treatment with 7.5 μM TF3 had no significant effect on the protein level of p-Histon H2A.X (Ser139) (*p* > 0.05). Treatment with 7.5 μM cisplatin significantly upregulated the protein level of p-Histon H2A.X (Ser139) (*p* < 0.05). The protein level of p-Histon H2A.X (Ser139) were significantly higher in both cells subjected to combination treatment than in untreated control cells and cells treated with either agent alone (*p* < 0.05). The results of Western blot analysis and ELISA assay indicated that TF3 can enhance DNA damage induced by cisplatin in ovarian cancer cells.

### 2.4. TF3 Synergistically Inhibited Ovarian Cancer Cells with Cisplatin by Reducing glutathione (GSH) Levels in the Cells

**Figure 4:**
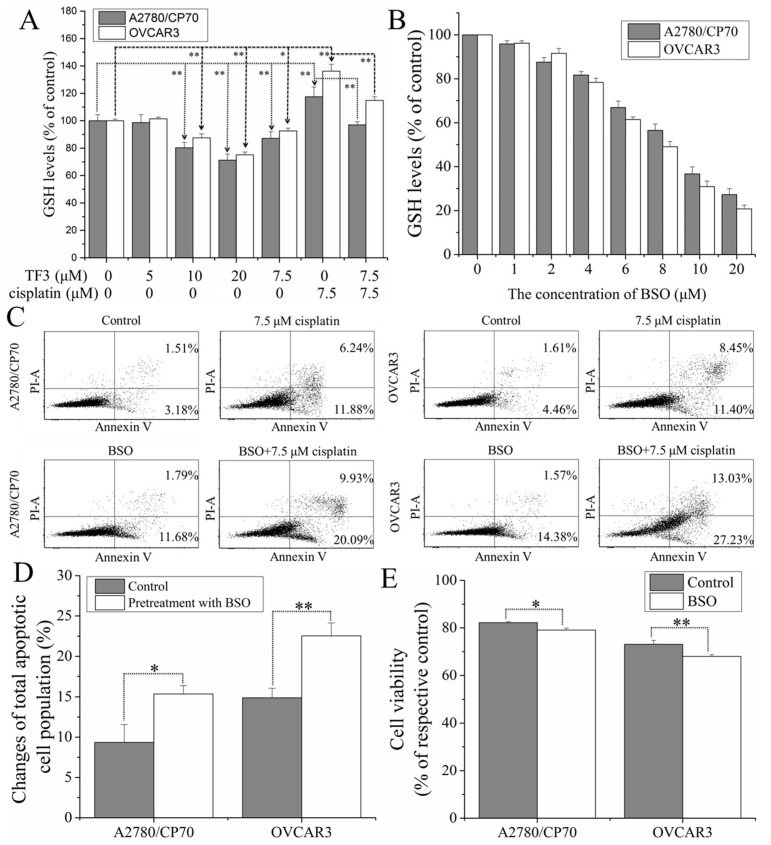
Treatment with TF3 potentiated inhibitory effect of cisplatin against ovarian cancer A2780/CP70 and OVCAR3 cells via reducing GSH levels. (**A**) The effect of TF3, cisplatin, or combination treatment on the GSH levels in ovarian cancer cells. The statistical difference analysis was performed between the groups at both ends of the broken lines with arrows; (**B**) treatment with BSO decreased the GSH levels in ovarian cancer cells in a dose-dependent manner.; (**C**) pretreatment with 2.0 μM BSO enhanced the pro-apoptotic effect of 7.5 μM cisplatin on ovarian cancer cells; (**D**) the changes of total apoptotic cell population induced by 7.5 μM cisplatin in control cells and cells pretreated with 2.0 μM BSO were expressed as quantification histograms with error bars; (**E**) pretreatment with 2.0 μM BSO enhanced the inhibitory effect of 7.5 μM cisplatin on the viability of ovarian cancer cells. Results are expressed as mean ± SD from three independent experiments. Significant differences among different treatments are marked with * (*p* < 0.05) and ** (*p* < 0.01).

To verify whether GSH was involved in the synergistic inhibitory effect of TF3 and cisplatin against ovarian cancer A2780/CP70 and OVCAR3 cells, the effect of TF3 on GSH levels and the effect of GSH on cell sensitivity to cisplatin were investigated. As shown in Figure 4A, treatment with TF3 decreased the GSH levels in both cells in a dose-dependent manner. Treatment with 7.5 μM cisplatin significantly increased the GSH levels in both cells (*p* < 0.01). GSH level in cells treated with combined 7.5 μM TF3 and cisplatin was significant lower than that in cells treated with 7.5 μM cisplatin (*p* < 0.01).

Buthionine sulphoximine (BSO), an irreversible inhibitor of γ-glutamylcysteine synthetase, was used to decreased GSH levels in cells. As shown in Figure 4B, treatment with BSO decreased the GSH levels in both cells in a dose-dependent manner. The reduced GSH levels induced by treatment with 2.0 μM BSO had no significant difference with that induced by treatment with 7.5 μM TF3, so that BSO at the concentration of 2.0 μM was used to investigate the effect of the reduced GSH levels on cell sensitivity to cisplatin. As shown in Figure 4C,D, Pretreatment with BSO significant increased the percentage of total apoptotic cells (*p* < 0.01 or 0.05). As shown in Figure 4E, BSO significantly enhanced the inhibitory effect of cisplatin against cell viability (*p* < 0.01 or 0.05). The results of flow cytometry analysis and cell viability assay indicated that the reduced GSH levels could enhance the sensitivity of both cells to cisplatin.

Taken together, treatment with TF3 potentiated the inhibitory effect of cisplatin against ovarian cancer A2780/CP70 and OVCAR3 cells by reducing GSH levels in both cells.

### 2.5. TF3 Upregulated the Protein Expression of CTR1 in Ovarian Cancer Cells

**Figure 5:**
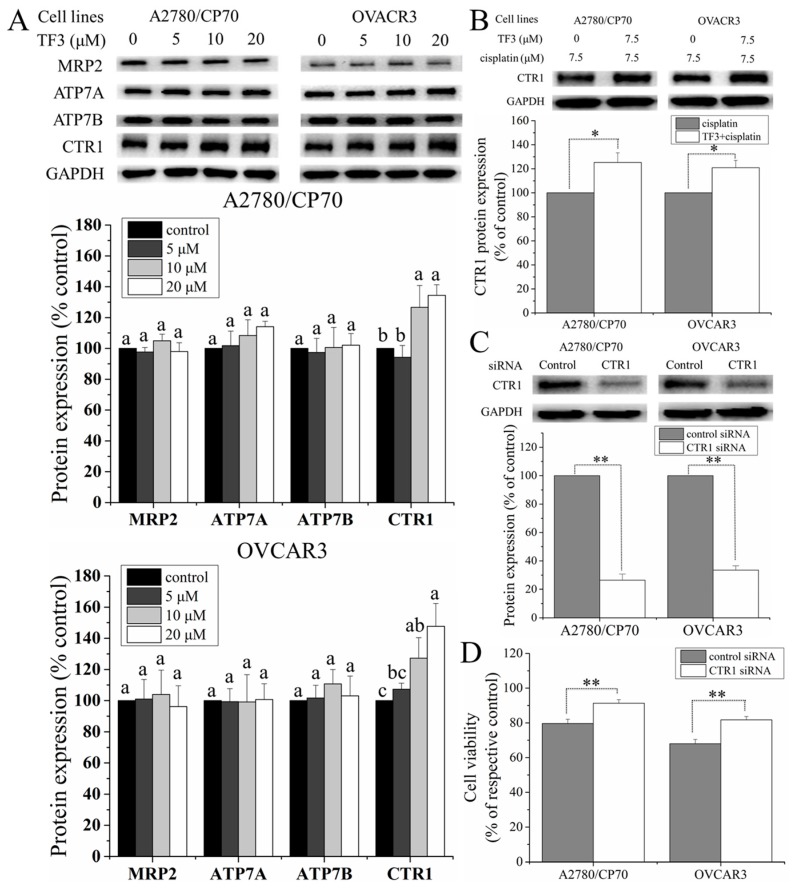
Treatment with TF3 potentiated inhibitory effect of cisplatin against ovarian cancer A2780/CP70 and OVCAR3 cells via upregulating CTR1 protein expression. (**A**) The effect of TF3 at the designated concentrations on the protein levels of MRP2, ATP7A, ATP7B and CTR1 in ovarian cancer cells; (**B**) treatment with 7.5 μM TF3 upregulated CTR1 protein levels in ovarian cancer cells pretreated with 7.5 μM cisplatin; (**C**) transfection with CTR1 siRNA decreased CTR1 protein levels in ovarian cancer cells; (**D**) transfection with CTR1 siRNA enhanced the resistance of ovarian cancer cells to 7.5 μM cisplatin. Results are expressed as mean ± SD from three independent experiments. Significant differences among different treatments are marked with different letters (*p* < 0.05), * (*p* < 0.05) and ** (*p* < 0.01).

Since cisplatin transporters can mediate the resistance of ovarian cancer cells to cisplatin, the protein levels of MRP2, ATP7A, ATP7B, and CTR1 in ovarian cancer A2780/CP70 and OVCAR3 cells were evaluated by Western blot analysis. As shown in Figure 5A, treatment with TF3 had no significant effect on the protein levels of MRP2, ATP7A, and ATP7B (*p* > 0.05) and upregulated the protein level of CTR1 in a dose-dependent manner. We further evaluated the effect of TF3 on the CTR1 protein level in cells treated with cisplatin. As shown in Figure 5B, treatment with 7.5 μM TF3 significantly upregulated the CTR1 protein level in cells treated with 7.5 μM cisplatin (*p* < 0.05). To further verify the correlation between CTR1 and cisplatin sensitivity, CTR1 in both cells were knocked down by transfection with CTR1 siRNA (Figure 5C) and then subjected to cell viability assay. As shown in Figure 5D, transfection with CTR1 siRNA significantly attenuated the inhibitory effect of 7.5 μM cisplatin on the cell viability of both cells (*p* < 0.01), indicating that the protein level of CTR1 mediated cisplatin resistance of both cells.

Taken together, treatment with TF3 could enhance the sensitivity of ovarian cancer A2780/CP70 and OVCAR3 cells to cisplatin by upregulating CTR1 protein expression in the cells.

## 3. Discussion

Cisplatin is one of the widely used chemotherapeutic drugs for cancer therapy. The resistance of ovarian cancer to cisplatin is a pivotal cause leading to its high lethal rate. The resistance to cisplatin is associated with reduced intracellular cisplatin accumulation, resulting from impaired intake, enhanced efflux, and increased sequestration of cisplatin. Thus, increasing intracellular cisplatin accumulation is a good way to overcome drug resistance. The combination therapy based on chemotherapeutic drugs and natural compounds has been reported to increase intracellular cisplatin accumulation by modulating cisplatin transport. EGCG enhances cisplatin sensitivity by regulating expression of CTR1 in ovarian cancer cells [25] and lung cancer cells [26]. The combination of glycyrrhizin and lamivudine attenuates the resistance of hepatocellular cancer cells to cisplatin through the inhibition of MRPs [27]. A reduction of intracellular GSH levels decreases the sequestration of cisplatin [28]. In the present study, we found that TF3 potentiates the inhibitory effect of cisplatin against ovarian cancer cells by modulating cisplatin transport and GSH.

In the present study, an MTS assay was first used to evaluate the synergistic inhibitory effect of TF3 and cisplatin against ovarian cancer A2780/CP70 and OVCAR3 cells. The combination treatment CI values were less than 1.0, indicating that the inhibitory effect was synergistic. Since the CI values of combined TF3 and cisplatin (7.5 μM:7.5 μM) were the lowest, TF3 and cisplatin at a ratio of 7.5 μM:7.5 μM was used for further study. Cisplatin exerted an antitumor effect mainly by inducing DNA damage. Furthermore, we determined the content of Pt and DNA-Pt adducts and DNA damage in ovarian cancer A2780/CP70 and OVCAR3 cells. Treatment with 7.5 μM TF3 significantly increased the intracellular accumulation of Pt and DNA-Pt adducts in both cells (*p* < 0.05). Treatment with TF3 enhanced DNA damage induced by cisplatin in both cells. The synergistic inhibitory effect of TF3 and cisplatin against ovarian cancer cells was involved in increased intracellular cisplatin accumulation. It has been reported that EGCG increases intracellular cisplatin accumulation by regulating the expression of CTR1 in ovarian cancer cells [25]. TF3 derives from EGCG. The same function groups of TF3 and EGCG might exert a synergistic inhibitory effect by increasing intracellular cisplatin accumulation in ovarian cancer cells.

GSH is a thiol-containing tripeptide consisting of glutamate, cysteine, and glycine. GSH is believed to be the major cellular target of cisplatin, which inactivates cisplatin by binding to it irreversibly. Cisplatin-resistant ovarian cancer cells often show elevated levels of cellular GSH [29,30,31]. In the present study, we found that treatment with TF3 decreased GSH levels and attenuated the elevation of GSH levels induced by cisplatin in ovarian cancer A2780/CP70 and OVCAR3 cells. Depletion of GSH enhanced the cytotoxicity of cisplatin against both cells. Taken together, the combined treatment with TF3 sensitized ovarian cancer cells to cisplatin by decreasing the cellular GSH levels. GSH is an important antioxidant that protects important cellular components from reactive oxygen species. It has been reported that TF3 inhibits human squamous carcinoma HSC-2 cells by inducing oxidative stress and reducing GSH levels [32]. Theaflavins induce oxidative stress and deplete GSH in human squamous carcinoma HSC-2 and CAL27 cells [33,34]. TF3 might decrease the GSH levels in ovarian cancer cells by inducing oxidative stress and further sensitize ovarian cancer cells to cisplatin.

Cisplatin transporters are important mediators of cellular uptake and efflux modulating the intracellular cisplatin accumulation, which determines the cytotoxicity of cisplatin [35]. In the present study, cisplatin transporters, including MRP2, ATP7A, ATP7B, and CTR1, were determined by Western blot analysis. Treatment with TF3 upregulated the protein levels of CTR1 in ovarian cancer A2780/CP70 and OVCAR3 cells, but had no significant effect on the protein levels of MRP2, ATP7A and ATP7B (*p* > 0.05). Treatment with TF3 also upregulated the protein levels of CTR1 in both cells treated with cisplatin. Furthermore, knockdown of CTR1 with siRNA significantly attenuated the inhibitory effect of cisplatin on the viability of both cells. CTR1 is a major transmembrane protein involving the uptake of cisplatin [36]. Enhanced expression of CTR1 sensitizes cancer cells to cisplatin by increasing cisplatin uptake [37]. The high expression level of CTR1 was found to be a prognostic factor for the improved survival of patients with advanced ovarian cancer [38]. Upregulated expression of CTR1 induced by TF3 enhances the sensitivity of ovarian cancer cells to cisplatin, and the upregulation of CTR1 induced by EGCG mediates cisplatin sensitivity in ovarian cancer cells [25].

## 4. Materials and Methods 

### 4.1. Cell Culture and Reagents

Human ovarian cancer cell lines A2780/CP70 and OVCAR3 were obtained from Βing-Hua Jiang at West Virginia University. The cells were cultured in RPMI-1640 medium (Sigma, St Louis, MO, USA) supplemented with 10% fetal bovine serum (Invitrogen, Rockford, IL, USA), 1% penicillin (100 U/mL), streptomycin (10 μg/mL), and amphotericin-B (250 μg/mL) (Sigma, St Louis, MO, USA) at 37 °C with 5% CO_2_ in a humidified incubator. TF3 monomers were isolated and purified using a previously established method [39]. Cisplatin was purchased from Sigma-Aldrich (Sigma, St Louis, MO, USA). TF3 and cisplatin were prepared in distilled water and stored at −20 °C. CTR1 siRNA and control siRNA were purchased from Santa Cruz Biotechnology (Danvers, MA, USA).

### 4.2. Cell Viability Assay

Cells were seeded in 96-well plates at 2 × 10^4^ cells per well and incubated for 12 h. The medium was replaced by treatment medium. After 24 h of treatment, CellTiter 96^®^ Aqueous One Solution Cell Proliferation Assay kit (Promega, St Louis, MO, USA) was used to determine cell viability. Cell viability was normalized by that of control cells for analysis.

### 4.3. Measurement of Intracellular Pt Accumulation

Cells were seeded in 6 cm dishes at 1 × 10^6^ cells per dish and incubated for 12 h. The medium was replaced by treatment medium. After 6 h of treatment, the cells were washed with phosphate buffered solution (PBS) to remove free cisplatin and harvested by trypsin. The protein concentrations were determined with BCA Protein assay kit (Pierce, St Louis, MO, USA). Total Pt content accumulated in cells was determined by Element 2 inductively coupled plasma mass spectrometry (ICP-MS) (Thermo Fisher, Waltham, MA, USA). To measure the content of Pt binding to DNA, DNA was extracted using PureLink™ Genomic DNA Mini Kit (Invitrogen, Rockford, IL, USA), according to the manufacturer’s protocol. The quantitation of DNA was measured using a Qubit Fluorometer (Invitrogen, Rockford, IL, USA). The samples were then digested in 5% nitric acid and measured by ICP-MS.

### 4.4. GSH Assay

Cells were seeded in 96-well plates at 2 × 10^4^ cells per well and incubated for 12 h. The medium was replaced by treatment medium. After 24 h of treatment, GSH levels in cells were detected using a GSH-Glo™ Glutathione Assay kit (Promega, St Louis, MO, USA) according to the manufacturer’s instructions. GSH levels were normalized by total protein levels and were expressed as percentage of the untreated control. The total protein levels were measured with a BCA assay kit.

### 4.5. Flow Cytometric Analysis of Apoptotic Cells

Cells were seeded in 6 cm dishes at 1 × 10^6^ cells per dish and incubated for 12 h. The medium was replaced by treatment medium. After 24 h of treatment, the cells were washed with PBS and harvested by trypsin. Cells were suspended in binding buffer and then stained with Alexa Fluor 488Annexin V and propidium iodide (PI) for 15 min. The cells were stained and then analyzed via flow cytometry (FACSCalibur system, BD Biosciences, Franklin Lakes, NJ, USA).

### 4.6. Cell-Based Phosphorylation ELISA Assay

Cells were seeded in 96-well plates at 2 × 10^4^ cells per well and incubated for 12 h. The medium was replaced by treatment medium. After 24 h of treatment, the treatment medium was removed. The amounts of phosphorylated Histone H2A.X were detected using a Phospho-H2A.X Cell-Based Phosphorylation ELISA Kit (LSBio, Seattle, WA, USA) according to the manufacturer’s instructions. The amounts of phosphorylated Histone H2A.X were normalized by total protein levels and were expressed as percentage of the untreated control. The total protein levels were measured with a BCA assay kit.

### 4.7. Western Blotting

Cells were seeded in 6 cm dishes at 1 × 10^6^ cells per dish and incubated for 12 h. The medium was replaced by treatment medium. After 24 h of treatment, cells were lysed using M-PER Mammalian Protein Extraction Reagent (Pierce, St Louis, MO, USA) supplemented with Halt™ Protease and Phosphatase Inhibitor Single-Use Cocktail (Life Technologies, Grand Island, NY, USA). The protein levels were determined using BCA Protein assay kit. After boiling for 6 min in loading buffer (Bio-Rad, Hercules, CA, USA), equal amounts of protein were subjected to sodium dodecyl sulfate polyacrylamide gel electrophoresis. The separated proteins were transferred onto a nitrocellulose blotting membrane. The membrane was blocked with 5% nonfat dried milk in Tris Buffered Saline (Bio-Rad, Hercules, CA, USA) containing 0.1% Tween-20 (TBST) at room temperature for 1 h and subsequently incubated with specific primary antibodies overnight at 4 °C. The membrane was washed three times (10 min each) with TBST and then incubated with an appropriate secondary antibody conjugated with horseradish peroxide for 1 h at room temperature. After three 10 min washes with TBST, antigen–antibody complex in each blot was visualized with Super Signal West Dura Extended Duration Substrate (Life Technologies, Grand Island, NY, USA) and ChemiDoc™ MP System (Bio-Rad, Hercules, CA, USA). Protein bands were quantitated with NIH ImageJ software and normalized by GAPDH bands for analysis.

### 4.8. Transfection with Small Interfering RNA (siRNA)

Cells were seeded in 6 cm dishes at 5 × 10^5^ cells per dish and incubated for 12 h. Then, CTR1 siRNA or control siRNA were transfected into the cells using Lipofectamine 2000 transfection reagent (Invitrogen, Rockford, IL, USA) according to the manufacturer’s instruction. Then the transfected cells were used for cell viability assay and Western blotting analysis. 

### 4.9. Statistical Analysis

The data were expressed as mean ± standard deviations. A least significant difference test was used to analyze multiple comparisons. A Student’s *t*-test was used to analyze the statistical difference between two groups. Statistically significant difference and highly significant difference were presented as *p* < 0.05 and *p* < 0.01, respectively.

## 5. Conclusions

In conclusion, our study revealed that TF3 potentiated the inhibitory effect of cisplatin against ovarian cancer A2780/CP70 and OVCAR3 cells by increasing the accumulation of intracellular Pt and DNA-Pt adducts. Treatment with TF3 downregulated cellular GSH levels and upregulated the protein expression of CTR1, resulting in elevated intracellular cisplatin accumulation. TF3 was a potential agent to reduce the side effect of cisplatin and overcome the cisplatin resistance of ovarian cancer cells.
